# From Tumor Biology to Clinical Perspectives: Novel Biomarkers and Therapeutic Insights in Gastric Cancer

**DOI:** 10.32604/or.2026.083832

**Published:** 2026-09-14

**Authors:** Xiya Cheng, Yunshu Ma, Jinglu Yan, Yizhe Zhang, Riguge Su, Xiaoming Tao, Jing Zhao, Peizhun Du

**Affiliations:** 1 Department of Medical Affairs, Huadong Hospital, Fudan University, Shanghai, China; 2 Hepatobiliary Surgery, Department of General Surgery, Huashan Hospital & Cancer Metastasis Institute, Fudan University, Shanghai, China; 3 Department of Digestive Diseases, Huadong Hospital, Fudan University, Shanghai, China; 4 Department of Neurology, Huashan Hospital, Fudan University, Shanghai, China; 5 Department of Endocrinology, Huadong Hospital, Fudan University, Shanghai, China; 6 Gastrointestinal Surgery, Department of General Surgery, Huashan Hospital, Fudan University, Shanghai, China

**Keywords:** Gastric cancer, genetic and epigenetic alterations, tumor microenvironment, microbiota-inflammation interactions, novel biomarkers

## Abstract

Gastric cancer (GC) is a leading cause of cancer-related mortality worldwide. Accurate early detection, timely diagnostic stratification, and robust prognostic risk assessment are essential for optimizing clinical outcomes and improving survival duration. However, conventional serological biomarkers demonstrate limited diagnostic performance owing to suboptimal sensitivity and specificity, while standard chemotherapy and targeted therapies provide only modest survival benefits in GC. Marked inter- and intratumoral heterogeneity further characterizes GC as a biologically complex and treatment-resistant malignancy. To date, significant progress has been made in comprehensively delineating the complex molecular pathogenesis of GC, providing a strong rationale for the development of novel biomarkers and promising therapeutic targets. The aim of this review is to systematically summarize recent advances ranging from aberrant genetic and epigenetic alterations; dysregulation of oncogenic signaling pathways; sophisticated crosstalk within the tumor microenvironment; intricate microbiota-inflammation interactions; fundamental discoveries to potential clinical translation, including novel biomarkers for early detection, diagnosis and prognostic stratification; and emerging therapeutic strategies for GC. Collectively, this work provides a conceptual framework for advancing precision medicine in GC.

## 1 Introduction

Gastric cancer (GC) remains a major global health challenge. According to the latest GLOBOCAN 2024 statistics, there were an estimated 980,000 new cases and 642,000 deaths worldwide, making it the fifth most frequently diagnosed cancer and the fifth leading cause of cancer-related mortality [1]. Despite steady improvements in surgical techniques and conventional chemotherapy regimens, the overall prognosis for patients with advanced GC remains poor, largely due to profound intra- and intertumoral heterogeneity. The classical Correa cascade provides a fundamental histopathological framework for understanding the stepwise progression from chronic gastritis to invasive adenocarcinoma [2]. At the molecular level, GC is driven by a highly complex and dynamic interplay of genetic mutations, epigenetic reprogramming, tumor microenvironment evolution, and microbiota-inflammation interactions.

In recent years, unprecedented advances in multi-omics and high-resolution sequencing technologies have enabled the dissection of the multilayered molecular pathogenesis underlying GC progression and therapeutic resistance. For example, single-cell RNA sequencing (scRNA-seq) and spatial transcriptomics have revolutionized our understanding of the highly plastic ecosystem within the tumor microenvironment (TME) [3]. We now recognize that GC progression is strongly influenced by immunosuppressive crosstalk orchestrated by cancer-associated fibroblasts (CAFs), tumor-associated macrophages (TAMs), regulatory T cells (Tregs), exhausted CD8^+^ T cells, and local microbial communities. These insights have, in turn, provided a strong biological rationale for the identification, validation, and clinical translation of next-generation biomarkers and actionable therapeutic targets.

Concurrently, the clinical management of GC is undergoing a paradigm shift toward biomarker-guided precision medicine. The continuous discovery of novel non-invasive liquid biopsy biomarkers—ranging from circulating tumor DNA (ctDNA) and circular RNAs to extracellular vesicles (EVs)—has demonstrated significant potential for early detection, accurate diagnostic and prognostic stratification, and real-time monitoring of disease progression in GC [4,5,6]. Cancer treatment is moving toward an era of precision oncology. Targeted therapies, pioneered by trastuzumab for HER2-positive tumors, have revolutionized the treatment paradigm from organ-based to biomarker-driven selection [7]. Recently, the advent of immune checkpoint inhibitors, antibody-drug conjugates (ADCs), bispecific T-cell engagers (BiTEs), and chimeric antigen receptor (CAR) T-cell therapies has further diversified the oncological armamentarium, offering unprecedented opportunities for treating refractory malignancies including GC. Nowaday, the clinical success of targeted therapies and immunotherapies, has profoundly improved the first-line therapeutic landscape of GC.

In this comprehensive review, we systematically summarize the latest breakthroughs in GC biology and clinical oncology. Specifically, this review aims to integrate current evidence on the molecular pathogenesis, tumor microenvironment, microbiota–inflammation axis, emerging biomarkers, and biomarker-guided therapies in GC, and to clarify their implications for precision diagnosis and individualized treatment. We first examine the complex molecular pathogenesis driven by dysregulated signaling cascades, genomic instability, epigenetic alterations, and RNA modifications. Afterward, we explore the sophisticated regulation of the TME and the pivotal microbiota-inflammation axis. We then discuss the emergence of novel liquid biopsy biomarkers and multi-omics panels for precision diagnostics. Finally, we review the current landscape and future directions of biomarker-guided therapeutic strategies. This work provides a hierarchical framework that links molecular mechanisms to cellular behaviors and, ultimately, to clinical implications in GC.

## 2 Complex Molecular Pathogenesis in GC

Gastric carcinogenesis is a multistep and multifactorial process. The classical Correa cascade delineates the sequential progression from chronic gastritis to atrophic gastritis, intestinal metaplasia, dysplasia, and ultimately invasive carcinoma, providing a fundamental histopathological framework [8]. This process involves complex molecular mechanisms, including genetic mutations, genomic instability, epigenetic alterations, and activation of oncogenic signaling pathways. These aberrations confer marked tumor heterogeneity and drive cancer initiation and progression. Understanding this complex pathogenesis is essential for precision subtyping and the discovery of novel biomarkers and therapeutic targets in GC.

### 2.1 Genetic Alterations and Dysregulated Oncogenic Pathways

GC is characterized not only by recurrent driver mutations but also by a multi-pathway activation pattern, in which multiple oncogenic cascades are simultaneously engaged. The Cancer Genome Atlas (TCGA) classified GC into four principal molecular subtypes—Epstein–Barr virus-positive (EBV), microsatellite instability (MSI), genomically stable, and chromosomal instability (CIN) [9]. CIN tumors are enriched in *TP53* alterations and receptor tyrosine kinase (RTK) amplification, whereas genomically stable tumors more often harbor abnormalities in cell adhesion and cytoskeletal regulation, including *CDH1* and *RHOA* alterations, as well as rearrangements involving CLDN18–*ARHGAP26* or *ARHGAP6* fusions [9]. EBV-positive and MSI tumors are more frequently associated with hypermutation, *PIK3CA* mutations, and *ARID1A* loss [9,10,11].

It has been well documented that loss-of-function *TP53* mutations impair genomic surveillance and facilitate chromosomal instability, thereby creating a permissive environment for additional oncogenic events [9]. In addition, several growth-promoting pathways are frequently activated, particularly the RTK/RAS/RAF/MEK/ERK and PI3K/AKT/mTOR cascades [9,11,12]. Amplification or activation of upstream receptors such as *ERBB2*, *EGFR*, *MET*, and *FGFR2* sustains downstream proliferative and survival signaling, promoting cell growth, resistance to apoptosis, and metabolic adaptation [9,11]. The Wnt/β-catenin pathway is another key oncogenic pathway in gastric carcinogenesis. Mutations in *APC* or inactivation of negative regulators such as *AXIN* can lead to abnormal nuclear accumulation of β-catenin, which activates transcriptional programs associated with stemness, proliferation, and cellular plasticity [13]. The PI3K/AKT/mTOR pathway, driven by *PIK3CA* mutations or *PTEN* loss, enhances survival signaling, metabolic reprogramming, and treatment resistance in GC [9,11].

Disruption of epithelial cohesion represents another major route of tumorigenesis. Germline *CDH1* mutations establish E-cadherin loss as a central mechanism in hereditary diffuse GC [14], and similar defects are also observed in sporadic diffuse-type tumors. In the genomically stable subtype of GC, concurrent *CDH1* and *RHOA* alterations impair cell-cell adhesion and cytoskeletal regulation, facilitating diffuse infiltration and invasive growth [9]. In addition, recurrent *ARID1A* mutations link oncogenic signaling to chromatin remodeling and reshape transcriptional programs involved in differentiation, DNA repair, and stress responses, particularly in the EBV-positive GC subtype [9,15].

Clinically, some of these genetic alterations are already actionable. *ERBB2* amplification has established therapeutic relevance and is targetable with trastuzumab, while CLDN18.2 targeting with zolbetuximab has demonstrated promising clinical efficacy in advanced GC.

### 2.2 Epigenetic Alterations and RNA Modifications in GC Progression

Epigenetic alterations and RNA modifications play key roles in regulating gene expression without changing DNA or RNA sequences. These modifications are highly dynamic and reversible. DNA methylation, histone modifications, and chromatin remodeling represent the major forms of epigenetic regulation. RNA modifications mainly include N^6^-methyladenosine (m^6^A), 5-methylcytosine (m^5^C), and N^4^-acetylcytidine (ac^4^C). Collectively, these molecular alterations have significant effects on GC cellular functions, including malignant proliferation, epithelial-mesenchymal transition (EMT), metastasis and invasion, metabolic reprogramming, and therapy resistance.

#### 2.2.1 Epigenetic Silencing and Early Initiation of GC

Promoter hypermethylation-mediated silencing of tumor suppressor genes is a critical driver of tumorigenesis in the early stages of GC development. Promoter hypermethylation of genes such as *p16*, *MLH1*, and *APC* is commonly observed in precancerous lesions, including chronic atrophic gastritis and intestinal metaplasia [16,17]. For example, the *CDH1* promoter is methylated in 30–50% of precancerous lesions, leading to disruption of cell adhesion [18]. *Helicobacter pylori* infection activates *DNMT1* and NF-κB signaling pathways, triggering genome-wide aberrant DNA methylation and establishing an “epigenetic memory” that can persist even after pathogen eradication, thereby facilitating tumor initiation [19,20,21]. Additionally, the EBV-positive GC subtype exhibits an extreme CpG island methylator phenotype, characterized by hypermethylation of nearly 20% of promoter CpG islands. EBV-induced hypermethylation silences key tumor suppressor genes and differentiation factors, thereby promoting a highly proliferative and poorly differentiated phenotype that favors early tumorigenesis in GC [9,22]. Promoter hypermethylation of genes such as *DAPK*, *CDH1*, *GSTP1*, *p15*, and *p16* can be detected in serum from GC patients, suggesting their potential as biomarkers [23]. Recently, methylated ctDNA has become a major focus in the development of novel GC biomarkers [24,25,26].

#### 2.2.2 Epigenetic Modifications Regulate Malignant Cell Proliferation, Metastasis, and Invasion of GC

Dysregulation of chromatin remodeling factors spans the entire trajectory of GC, from local invasion to systemic dissemination. *CHD4*, a core subunit of the Mi-2/NuRD complex, exhibits non-canonical functions by interacting with *MYH9* to induce GSK3β degradation. This stabilizes β-catenin, leading to sustained activation of Wnt signaling and EMT in GC [27].

M^6^A modification is the most prevalent RNA epigenetic modification, orchestrated by a range of writers, readers, and erasers. *METTL3*, a well-documented m^6^A writer, enhances the stability of the oncogenic *CENPF* mRNA transcript, thereby activating the FAK/MAPK signaling pathway and promoting GC metastasis [28]. By contrast, another m^6^A writer, *METTL14*, suppresses GC growth and invasion by inducing m^6^A modification of *circORC5*, thereby regulating the *miR-30c-2-3p*/*AKT1S1* axis [29]. M^6^A readers, including *YTHDF1/2/3*, *IGF2BP1/2/3*, and *HNRNPA2B1*, recognize methylation marks to determine RNA fate. For example, the m^6^A reader *YTHDF1* enhances the translation of m^6^A-modified *FZD7*, thereby activating the Wnt/β-catenin signaling axis and promoting malignant proliferation and metastasis in GC [30]. *ALKBH5* and *FTO* function as m^6^A erasers. *ALKBH5*-mediated demethylation of *WRAP53* reduces *RALBP1* expression and suppresses activation of the PI3K/AKT/mTOR pathway, thereby inhibiting GC metastasis [31].

In addition, m^5^C modification has been implicated in the regulation of alternative splicing. The RNA-binding protein *NONO* recruits *NSUN2* to *PTEN* pre-mRNA, altering its methylation pattern and leading to aberrant splicing and reduced expression of functional *PTEN*. This ultimately activates the PI3K/AKT signaling pathway and promotes proliferation, migration, and invasion in GC [32]. *NAT10*-mediated ac^4^C modification has also been reported to enhance the stability of the splicing factor *SRSF2* in GC, thereby promoting exon skipping of *YTHDF1* and generating oncogenic transcript variants, which in turn stimulate proliferation and metastasis [33,34].

#### 2.2.3 Epigenetic Alterations Modulate Metabolic Reprogramming of GC

Epigenetic modifications play critical roles in regulating metabolic reprogramming in GC. A recent study demonstrated that the synergistic effects of H3K27ac and H3K4me3 drive lncRNA *DLEU1* expression, which promotes glycolysis and activates *G6PD* to increase NADPH production via the *ASCC2*/*ALKBH3* axis. This enhanced NADPH production helps maintain redox homeostasis and supports metabolic adaptation [35]. P300-mediated H3K27 acetylation can also increase *METTL3* transcription, thereby promoting m^6^A modification of *HDGF* mRNA and enhancing its stability. The resulting nuclear *HDGF* subsequently activates *GLUT4* and *ENO2* expression, thereby increasing glycolysis in GC [36]. Beyond m^6^A, other RNA modifications also contribute to GC progression. *NSUN2*-mediated m^5^C modification enhances the stability of lncRNA *NR_033928*, which acts as a molecular scaffold to recruit the *IGF2BP3*/HUR complex and stabilize *GLS* mRNA, thereby promoting glutamine metabolism reprogramming and tumor growth in GC [37]. Natural product brusatol can inhibit glycolysis-driven H3K18la and H3K9la, thereby blocking GC progression [38]. Targeting epigenetic alterations to remodel metabolism offers a promising therapeutic strategy for GC.

#### 2.2.4 Epitranscriptomic Plasticity Contributes to Therapy Resistance in GC

RNA modifications confer high molecular plasticity to GC cells and have a crucial impact on chemoresistance. For example, the m^6^A methyltransferase *METTL3* enhances the stability of transcripts such as *ARF6* and *PARP1* through m^6^A modification, thereby promoting resistance to chemotherapeutic agents, including cisplatin and 5-fluorouracil (5-FU) in GC [39,40]. The m^6^A reader *HNRNPA2B1* can also induce chemoresistance by stabilizing lncRNA *NEAT1* and maintaining GC stemness [41]. By contrast, another m^6^A reader, *YTHDF2*, has been reported to suppress cancer stemness and improve chemosensitivity to oxaliplatin in GC by promoting the degradation of *ONECUT2* mRNA [42]. In addition, m^6^A demethylases (e.g., *ALKBH5*, *FTO*) and the m^5^C methyltransferase *NSUN2* are also involved in resistance regulation [43,44,45]. Knockdown of *NSUN2* can increase the sensitivity of GC cells to cisplatin and 5-FU [44].

Additionally, histone modifications can also contribute to therapy resistance in GC and have emerged as promising targets for improving treatment efficacy. The novel HDAC inhibitor chidamide, which specifically targets *HDAC3*, increases *HNF4A* acetylation levels and downregulates *TYMS*, thereby effectively reversing 5-FU chemoresistance in GC cells. *EZH2* is a well-known histone methyltransferase that catalyzes H3K27me3-mediated transcriptional repression. *EZH2* deficiency leads to loss of H3K27 methylation, triggering activation of Tfap2c and inducing aberrant squamous differentiation and chemotherapy resistance in GC [46].

**Figure 1:**
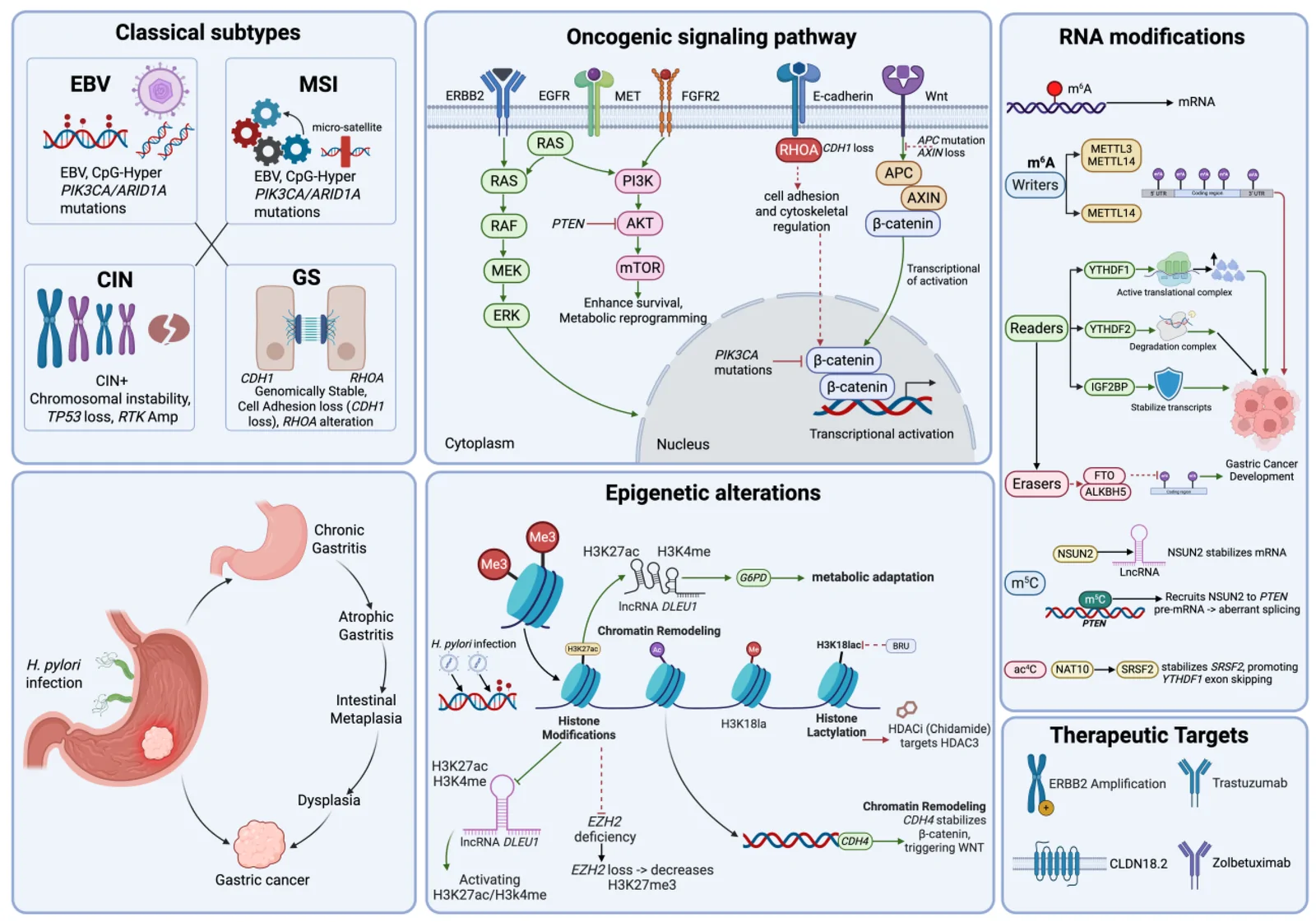
**Integrated molecular landscape of gastric cancer (GC) pathogenesis and therapeutic targets.** The schematic summarizes the major classical genomic subtypes, including Epstein–Barr virus (EBV)–positive tumors, microsatellite instability (MSI), chromosomal instability (CIN), and genomically stable (GS) GCs, and links *Helicobacter pylori*–driven chronic inflammation to the histological progression from gastritis to intestinal metaplasia, dysplasia, and carcinoma. The figure also highlights key oncogenic signaling cascades, epigenetic alterations, such as DNA methylation, histone modification, lactylation, and chromatin remodeling, together with RNA modifications including m6A, m5C, and ac4C, which play essential roles in GC progression. Clinically actionable targets, such as *ERBB2* amplification and CLDN18.2, are shown with representative antibodies trastuzumab and zolbetuximab. Created with BioRender.com. Abbreviations: CIN, chromosomal instability; EBV, Epstein-Barr virus; GS, genomically stable; MSI, microsatellite instability.

The schematic illustration in Fig. 1 depicts the complex pathogenesis of GC. Integrating GC molecular subtypes into clinical practice enables more refined prognostic stratification and facilitates the delivery of targeted therapies. Emerging epigenetic and epitranscriptomic markers—such as methylated ctDNA and RNA modifications—show promising potential as novel biomarkers. The reversibility of these epigenetic modifications further supports the development of novel therapeutics to overcome treatment resistance. However, prospective validation and standardized detection platforms are still required to enable their clinical translation in GC.

## 3 Sophisticated Regulations of TME in GC Progression

Accumulating evidence has demonstrated that the TME plays a critical role in GC. The TME is a complex ecosystem composed of diverse immune cells, stromal cells, endothelial cells, and the extracellular matrix (ECM). These components engage in intricate crosstalk, collectively promoting immunosuppression and immune evasion, and ultimately driving GC cell proliferation, metastasis, and therapeutic resistance.

### 3.1 Multifunctional Roles of CAFs in GC Development

CAFs can directly activate pro-oncogenic signaling pathways in GC cells by secreting factors such as IL-6, IL-8, and TGF-β [47,48]. Moreover, CAFs are key drivers of ECM remodeling, promoting tumor invasion and metastasis [49]. Recent large-scale scRNA-seq data have characterized CAF heterogeneity in GC and identified a distinct *INHBA*- and FAP-high CAF subpopulation associated with poor prognosis, as *INHBA* can increase FAP expression and induce collagen transcription to mediate stromal remodeling [50]. Lu et al. reported that CAF-secreted *SEMA7A* binds to *ITGB1* on GC cells, triggering *GAL3ST1*-mediated H3Y99 sulfation (H3Y99sulf), which recruits *KAT2A* to establish H3K56ac modification. This cascade activates the β-catenin pathway, driving EMT and metastasis [51]. Additionally, CAF-derived lactate induces increased H3K18la levels in GC cells, activating the *ASPM*/*NCAPG*/*STAT3* axis and upregulating PD-L1, which contributes to immunotherapy resistance [52]. Recent research has also revealed a unique nicotinamide “metabolic face-off” between macrophages and CAFs [53]. Specifically, macrophages are enriched in nicotinamide phosphoribosyltransferase (*NAMPT*), whereas CAFs highly express Notch-regulated nicotinamide N-methyltransferase (*NNMT*). This competitive expression of rate-limiting enzymes dynamically alters nicotinamide availability within the TME, which in turn directly influences the antitumor activity and exhaustion status of local CD8^+^ T cells. As a counter-regulatory mechanism, macrophages can release *NAMPT*-enriched EVs that inhibit *NNMT* transcription in CAFs via the *SIRT1*/NICD axis [53].

### 3.2 Complicated Crosstalk of TAMs in GC

TAMs, predominantly exhibiting M2-like polarization, are a major component of the TME in GC. They foster an immunosuppressive niche, promote therapeutic resistance, and accelerate cancer progression. Recent studies have identified *DKK1* as a key factor driving macrophage recruitment through binding to the *CKAP4* receptor on the macrophage surface, thereby activating the PI3K/AKT pathway to induce M2 polarization and suppress CD8^+^ T-cell and Natural Killer (NK) cell function in GC [54]. Blockade of *DKK1* not only reduces TAM infiltration but also synergizes with anti-PD-1 therapy, overcoming primary resistance [54]. Exosomes mediate bidirectional communication between GC cells and TAMs. GC cells highly express *SERPINE1* through autocrine *JAK2*/*STAT3* signaling and subsequently release *let-7g-5p*-enriched exosomes to macrophages, which potently drive M2 polarization by inhibiting *SOCS7* and activating *STAT3* [55]. Another study indicated that TAMs can release *MALAT1*-enriched exosomes to GC cells, activating the β-catenin/HIF-1α pathway to promote the Warburg effect, thereby enhancing tumor proliferation and chemotherapy resistance [56]. lncRNA NR-109 is highly expressed in TAMs and can block *FUBP1* degradation while activating c-Myc to promote M2-like macrophage polarization in GC [57]. Reciprocally, c-Myc can function as an upstream transcription factor to stimulate NR-109 expression, forming a positive feedback loop that drives pro-oncogenic M2 polarization in GC [57].

### 3.3 Oncogenic Functions of Tumor-Associated Neutrophils (TANs) and Neutrophil Extracellular Traps (NETs) in GC Aggravation

The role of TANs in GC has become increasingly prominent. Recent investigations have shown that microbial communities within the TME can recruit TANs. *Fusobacterium nucleatum*, enriched in GC tissues, can recruit large numbers of TANs by activating the IL-17/NF-κB/RelB pathway and inducing PD-L1 expression, leading to CD8^+^ T-cell exhaustion and immune evasion [58]. Polymorphonuclear (PMN) Myeloid-Derived Suppressor Cells (MDSCs) are often regarded as neutrophils in a pathological state and exhibit potent immunosuppressive functions. Their abundance in the TME is frequently associated with poor prognosis in GC [59]. Previous studies have shown that PMN-MDSCs exert dual pro-tumor effects by secreting the *S100A8/A9* heterodimer, which simultaneously affects GC cells and CD8^+^ T cells. *S100A8/A9* binds to *TLR4* on GC cells and activates the p38 MAPK/NF-κB pathway to increase *CXCL1* expression, thereby promoting PMN-MDSC recruitment. Meanwhile, *S100A8/A9* directly induces CD8^+^ T-cell exhaustion via the *TLR4*/AKT/mTOR pathway, suppressing T-cell glycolysis, proliferation, and production of TNF-α and IFN-γ [59].

Notably, overactivated neutrophils can release large amounts of DNA-rich NETs, which have emerged as an attractive research target in GC. The hypoxic TME in GC can recruit neutrophils and induce NET formation via the HMGB1/*TLR4*/p38 MAPK pathway, thereby promoting invasion, migration, angiogenesis, and tumor growth [60]. Mouse model studies indicate that infection-induced NET formation in peripheral blood and ascites may facilitate GC cell extravasation and dissemination to the liver and peritoneum [61]. Zhang et al. further demonstrated that activation of the TGF-β/Smad/LIF signaling axis is critical for mediating NET formation, thereby driving peritoneal metastasis of GC [61]. Targeting NET formation is therefore emerging as a promising strategy for suppressing GC metastasis [60].

### 3.4 T-Cell Dysfunction Exacerbates GC Progression

T cells serve as the primary effector cells of antitumor immunity, and their functional status directly influences tumor progression and immunotherapy outcomes. However, the immunosuppressive TME impairs T-cell activation, infiltration, and cytotoxic function, thereby reducing the efficacy of immunotherapies. As described above, CAFs, TAMs, TANs, NETs, and MDSCs are major cellular components that shape an immunosuppressive TME, promoting CD8^+^ T-cell exhaustion and suppressing antitumor immunity. In addition, metabolic competition between tumor cells and immune cells is a key mechanism underlying T-cell dysfunction. Gut microbial metabolites can also modulate the cytotoxic function of CD8^+^ T cells in GC. Yu et al. demonstrated that patients with GC commonly exhibit reduced abundance of butyrate-producing gut microbiota and decreased circulating butyrate levels [62]. Exogenous butyrate supplementation significantly enhances the antitumor cytotoxicity of CD8^+^ T cells by activating the *GPR109A*/*HOPX* signaling axis [62].

Tregs are critical drivers of immunosuppression and immune evasion in GC. GC-derived IL-33 enables mast cells to secrete IL-2, which induces Tregs to express *ICOS*. These *ICOS*^+^ Tregs can potently suppress CD8^+^ T-cell proliferation and effector molecule production, thereby promoting GC progression [63].

### 3.5 The Landscape of TME Spatiotemporal Evolution in GC

The rapid advancement of single-cell RNA sequencing (scRNA-seq) and spatial transcriptomics (ST) technologies has provided a high-resolution framework for dissecting cellular plasticity and the spatial evolution of the TME in GC [3]. In early EGC, single-cell atlases have revealed the enrichment of *CH25H*^+^ CD4^+^ T cells, *ADAMTSL2*^+^ endothelial cells, and other subsets. Notably, IL-33^+^ endothelial cells not only enhance angiogenesis but also directly upregulate *KRT17* expression in GC cells, thereby promoting tumor growth [64]. Spatial transcriptomics further optimizes the characterization of molecular dynamics with high spatial precision. Spatiotemporal multi-omics analyses of endoscopic submucosal dissection specimens from EGC have identified, for the first time, precancerous microenvironment (PMC_P) and PMC_2 cell subpopulations that drive carcinogenic initiation [65]. Combined with single-cell analysis, Zhang et al. demonstrated that GC stem cells originate from mature gastric chief cells through spasmolytic polypeptide-expressing metaplasia (SPEM) transdifferentiation, maintaining stemness and drug resistance via the iCAF-secreted *AREG*–*ERBB2* signaling axis and establishing an immune-evasive microenvironment [66]. Currently, the abundance of tertiary lymphoid structures (TLSs) has become a key indicator for assessing therapeutic efficacy. Wang et al. found that high TLS abundance and increased infiltration of intratumoral *CXCL13*^+^ *CD160*^+^ CD8^+^ T cells prior to treatment in advanced GC patients are significantly associated with a favorable treatment response [67]. Mechanistically, intracellular vitamin B6 in *CD160*^+^ CD8^+^ T cells may inhibit *MDM2*-mediated HIF-1α ubiquitination and degradation, leading to upregulation of *CXCL13* expression and promoting *CXCR5*^+^ B cell recruitment and TLS maturation [67].

Taken together, a comprehensive understanding of TME characteristics is of profound importance for developing novel therapeutic strategies that target not only GC cells but also the immunosuppressive microenvironment. These findings provide a solid theoretical foundation for therapeutic interventions, including targeting key stromal-immune axes (e.g., *DKK1*–*CKAP4*, *SEMA7A*–*ITGB1*, and NETosis pathways), reversing metabolic dysregulation through taurine and butyrate supplementation or *SLC6A6* inhibition, and leveraging spatial transcriptomic signatures alongside TLS-based stratification to guide personalized combination immunotherapies. Such integrated approaches hold promise for overcoming treatment resistance and improving clinical outcomes in GC.

**Figure 2:**
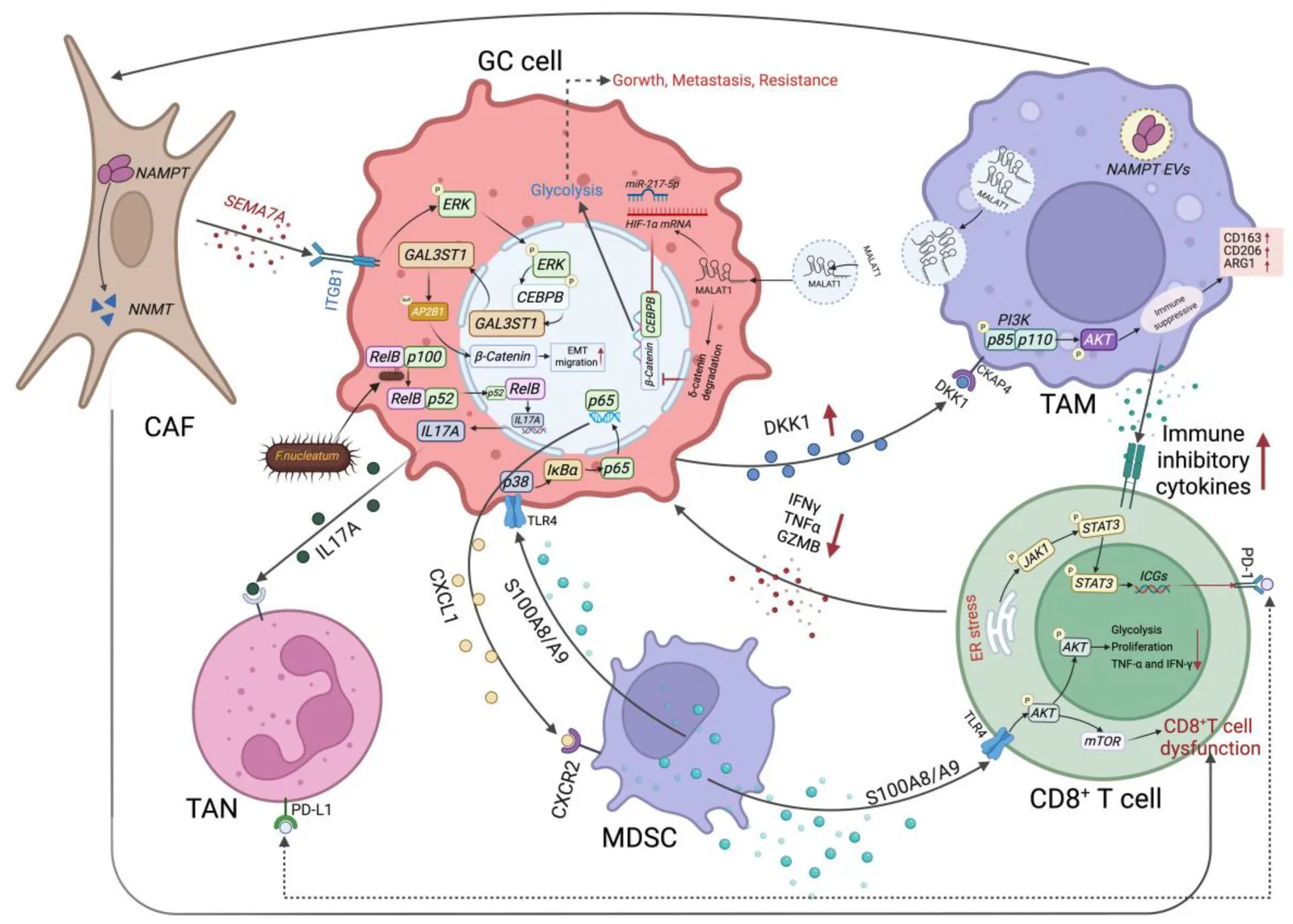
**Overview of cellular interactions in the GC tumor microenvironment.** This schematic illustrates the crosstalk between GC cells and major components of the tumor microenvironment, including CAFs, TAMs, TANs, MDSCs, and CD8^+^ T cells. These interactions activate multiple signaling pathways that promote tumor cell proliferation, migration, metastasis, metabolic reprogramming, immune suppression, and CD8^+^ T cell dysfunction. Created with BioRender.com. Abbreviations:GC, gastric cancer; CAF, cancer-associated fibroblast; TAN, tumor-associated neutrophil; MDSC, myeloid-derived suppressor cell; TAM, tumor-associated macrophage.

Fig. 2 delineates diverse ligand-receptor axes and secreted factors mediating crosstalk among GC cells, cancer-associated fibroblasts, myeloid suppressor cells and cytotoxic T lymphocytes, which collaboratively induce CD8^+^ T cell exhaustion and accelerate malignant progression.

## 4 The Microbiota-Inflammation Axis in GC Development

The microbiota-inflammation axis has emerged as a key factor in gastric carcinogenesis, integrating microbial dysbiosis, host immune responses, and tumor-promoting processes into a dynamic and interconnected network. Beyond the classical *H. pylori*-driven model, accumulating evidence highlights the contribution of a broader microbial ecosystem that collectively sustains chronic inflammation and promotes cancer development [68]. Notably, this axis spans multiple stages of GC, from early mucosal injury to disease progression and therapeutic response, with some microbiota-based interventions showing translational potential [69,70]. In this section, we summarize the initiation of dysbiosis-driven inflammation, the underlying molecular networks, key microbial drivers, and their clinical and therapeutic implications in GC.

### 4.1 Dysbiosis-Driven Inflammatory Initiation of Gastric Carcinogenesis

The intricate interplay between the gastric microbiota and chronic inflammation represents a fundamental axis in gastric carcinogenesis. This “inflammation-microbiota-cancer” axis is initiated by microbial dysbiosis, which disrupts gastric homeostasis and establishes a self-perpetuating cycle of mucosal injury and inflammatory response. Persistent inflammation, in turn, further reshapes the microbial community and reinforces dysbiosis [71]. In the highly acidic gastric environment, *H. pylori* remains the dominant pathogenic driver; however, emerging evidence implicates non-*H. pylori* microbiota in sustaining this inflammatory cascade and promoting premalignant progression, highlighting a shift from a single-pathogen model to a complex microbial ecosystem perspective [72,73]. This dysbiosis-driven chronic inflammation ultimately creates a procarcinogenic milieu that facilitates genomic instability and neoplastic transformation [74].

### 4.2 Molecular Signaling Networks Linking Microbiota and Inflammation

At the molecular level, the crosstalk between the microbiota and host inflammatory responses is orchestrated through a complex signaling network centered on the NF-κB pathway, which serves as a critical hub linking microbial dysbiosis to gastric tumorigenesis [75]. Specific microbial components and toxins activate inflammatory signaling cascades, including the NF-κB and MAPK pathways, leading to the transcription of pro-inflammatory cytokines that contribute to gastrointestinal carcinogenesis [74].

In parallel, additional signaling pathways, including *STAT3* and PI3K/AKT, are activated by microbial stimuli to regulate cell survival, proliferation, and inflammatory responses [76]. The *NLRP3* inflammasome serves as another key effector activated by bacterial virulence factors such as *H. pylori* CagA, leading to caspase-1-dependent maturation of IL-1β and IL-18 and amplification of inflammatory responses [77]. Systemic inflammatory indices, including the neutrophil-to-lymphocyte ratio (NLR) and systemic inflammation response index, are strongly associated with cancer cachexia in patients with GC [78].

Conversely, microbiota-derived metabolites, particularly short-chain fatty acids, exert anti-inflammatory effects by inhibiting histone deacetylases (HDACs), thereby suppressing NF-κB activation and preventing excessive inflammation [79]. Dynamic alterations in the microbiota during gastric carcinogenesis are associated with both epigenetic and genetic changes, including DNA methylation and *TP53* mutations [80].

Collectively, these pathways form a dynamic regulatory network through which the microbiota can both initiate and restrain inflammatory responses, thereby maintaining or disrupting gastric homeostasis.

### 4.3 Microbial Drivers and Pro-Tumorigenic Mechanisms

Bacteria often exhibit distinct pro-inflammatory and tumor-promoting mechanisms. *H. pylori* employs a sophisticated virulence arsenal: the CagA oncoprotein activates NF-κB and β-catenin signaling, while VacA induces epithelial vacuolation and disrupts autophagy. In addition, the HtrA polymorphism (171S/L) enhances cleavage of E-cadherin and occludin, thereby compromising epithelial barrier integrity [81,82,83]. *H. pylori*-secreted Gamma-Glutamyl Transferase (GGT) also depletes glutamine and α-KG in gastric epithelial cells and increases histone H3K9me3 and H3K27me3 levels. This epigenetic reprogramming activates the Wnt signaling pathway and contributes to gastric carcinogenesis [84]. *H. pylori* CagA can also upregulate transcription of the RNA demethylase *FTO* via *JUN*, leading to demethylation and stabilization of *HBEGF* mRNA, thereby facilitating EMT and metastasis [85]. Large-scale community-based eradication trials have shown that *H. pylori* infection eradication significantly reduces long-term GC risk, particularly in individuals without precancerous lesions [86,87].

Beyond *H. pylori*, several non-*H. pylori* commensals also function as direct contributors to carcinogenesis. Streptococcus anginosus activates the TMPC/ANXA2/MAPK pathway, promoting cell proliferation and epithelial barrier disruption [88]. In addition, the microbiota can modulate the TME in GC. Streptococcus anginosus has been shown to promote immune evasion through arginine-ornithine metabolism, thereby suppressing CD8^+^ T-cell infiltration [89], while *Fusobacterium nucleatum* further contributes to protumoral neutrophil recruitment via the IL-17/NF-κB/RelB axis [58]. These microbial interactions support a broader gastric microbial axis that sustains a pro-inflammatory network driving gastric carcinogenesis [90]. Probiotic and prebiotic interventions also show therapeutic potential. Engineered probiotics can alleviate inflammation by scavenging reactive oxygen species and modulating microbial composition [91], while prebiotics such as inulin enhance antitumor immunity [69].

### 4.4 EBV Infection Promotes GC Development

In addition to bacterial infection, EBV represents a distinct carcinogenic driver in GC. EBV-associated GC is characterized by widespread CpG island hypermethylation and recurrent *PIK3CA* and *ARID1A* alterations [15,92]. A recent study reported that EBV can upregulate *KDM5B* through cooperation between latent and lytic viral proteins, leading to transcriptional repression of the tumor suppressor *PLK2* and activation of the PI3K/AKT/mTOR pathway, thereby promoting malignant GC proliferation [93]. In parallel, EBV-encoded non-coding RNAs critically reshape the tumor microenvironment and epigenetic landscape. EBV-*circLMP2A* enhances hypoxia-driven angiogenesis via a HIF1α/*VEGFA* positive feedback loop, contributing to tumor vascularization and GC progression [94], while EBV-*circRPMS1* promotes GC growth and invasion by activating *METTL3*-dependent m6A modification [95]. Immune escape is another central hallmark of EBV-driven carcinogenesis, as viral miRNAs (*BART11* and *BART17-3p*) upregulate PD-L1 expression by targeting *FOXP1* and *PBRM1* [96], while *LMP2A* induces platelet aggregation and TGF-β release, thereby suppressing NK cell cytotoxicity [97]. EBV-associated GC is characterized by a highly inflamed tumor microenvironment with abundant CD8^+^ T-cell infiltration [98]. Consistent with this feature, EBV-positive GC demonstrates remarkable sensitivity to immune checkpoint blockade in both advanced and neoadjuvant settings [99,100].

### 4.5 Clinical Implications of the Microbiota-Inflammation Axis

Accumulating evidence increasingly highlights the prognostic and therapeutic relevance of microbiota-inflammation interactions. Microbiota-based signatures may provide valuable diagnostic and prognostic utility. For example, deep learning models incorporating oral taxa such as Aggregatibacter outperform conventional TNM staging in predicting GC prognosis [101]. Importantly, microbiota composition also influences therapeutic response, as prior antibiotic exposure has been shown to impair immunotherapy efficacy by promoting CD8^+^ T-cell exhaustion [102]. In addition, fecal microbiota transplantation strategies are gaining increasing attention. Clinical studies have demonstrated their safety and potential to overcome immunotherapy resistance [103]. Together, these findings underscore the translational potential of targeting the microbiota-inflammation axis as a promising complementary strategy for GC prevention and treatment.

## 5 Novel Biomarkers for Early Detection, Diagnosis, and Prognosis in GC

Early detection, accurate diagnosis, and precise prognostic prediction are the cornerstones for improving outcomes in GC. Traditional endoscopic screening is invasive, costly, and often inconvenient for patients. With the advent of high-throughput sequencing and multi-omics technologies, a growing number of non-invasive liquid biopsy biomarkers have emerged, demonstrating improved sensitivity and specificity. These novel biomarkers include proteins, circulating nucleic acids (DNA, miRNA, lncRNA, and circRNA), and EVs, which can be detected in both blood and gastric juice.

### 5.1 Protein Biomarkers

Conventional blood-based biomarkers such as CEA, CA19-9, and CA72-4 exhibit limited sensitivity and specificity in GC. Accordingly, numerous novel serum proteins have been extensively investigated as potential diagnostic biomarkers. Recent studies have reported that interleukin enhancer-binding factor 2 (*ILF2*) is markedly increased in the serum of GC patients and demonstrates strong diagnostic performance, with an Area Under the Curve (AUC) of 0.915 for GC versus healthy controls, 0.854 for GC versus benign gastric disease, and 0.888 for stage I GC versus benign gastric disease plus healthy controls—outperforming conventional blood-based indicators in the same cohort [104]. Similarly, insulin-like growth factor binding protein 7 (*IGFBP7*) has shown promise for early diagnosis, with an AUC of 0.773 (95% CI: 0.701–0.845) in early GC [105]. *SNCG* (γ-synuclein) is another promising biomarker for early detection in GC; it is significantly upregulated in both gastric juice and serum of GC patients, achieving an AUC of 0.923 with high sensitivity and specificity [106].

### 5.2 Circulating Tumor DNA (ctDNA) Biomarkers

ctDNA has emerged as an ideal liquid biopsy biomarker over the past decade, as it can reflect tumor-derived molecular alterations earlier than conventional diagnostic modalities.

Copy number variation (CNV) of the HER2 gene (also known as *ERBB2*) represents a frequent genomic abnormality in gastric carcinogenesis. Increased HER2 CNV can be detected in serum ctDNA from GC patients and can distinguish GC cases from healthy controls with a specificity of 98% [4]. ctDNA detection is also valuable for guiding therapeutic selection. In advanced GC, *EGFR*-amplified ctDNA is associated with better response to *EGFR* inhibitors, whereas *FGFR2* and *MET* co-amplification is associated with limited benefit from *EGFR* blockade [107]. Additionally, longitudinal postoperative ctDNA monitoring can detect GC recurrence earlier than radiographic evidence of relapse [108,109]. Methylated DNA is another promising liquid biopsy biomarker for GC. A landmark marker discovery study identified a three-marker plasma methylation panel consisting of *ELMO1*, *ZNF569*, and *C13orf18* (now standardized as *C13orf18*/*ZNF569*), which detected 86% of GC cases at 95% specificity [110]. Another plasma-based methylation panel combining *ELMO1*, *ZNF582*, and *TFPI2* has also been proposed for the detection of upper gastrointestinal cancers [111].

Additionally, serial monitoring of cfDNA methylation profiles has emerged as a powerful tool for tracking disease dynamics and guiding therapeutic decision-making. A recent pan-cancer methylation analysis of cfDNA demonstrated that a universal cancer-only methylation marker panel enables multi-organ cancer detection and longitudinal monitoring through simple blood-based assays, highlighting the clinical potential of integrating methylation-based liquid biopsy into routine surveillance protocols [112]. In GC, such dynamic cfDNA monitoring may facilitate real-time assessment of treatment response, early detection of molecular residual disease, and identification of emerging resistance, thus guiding adaptive treatment modifications throughout the course of GC progression.

### 5.3 Circulating Non-Coding RNAs as Biomarkers

Circulating non-coding RNAs mainly include circulating miRNAs, long non-coding RNAs (lncRNAs), and circular RNAs (circRNAs), which are stable in bodily fluids and have been widely investigated as diagnostic, prognostic, and therapeutic biomarkers in GC [113].

#### 5.3.1 Circulating RNAs as Promising Diagnostic Biomarkers in GC

For early GC detection, serum *miR-181* and *miR-652* both show promising diagnostic value, with AUCs of 0.820 and 0.842, respectively. When combined with the conventional marker CA72-4, the three-marker model achieved an AUC of 0.917 (95% CI: 0.856–0.975), with 92.5% sensitivity and 86.8% specificity [114]. An optimized three-miRNA signature (*miR-18a*, *miR-181b*, and *miR-335*) for early GC detection was established based on genome-wide expression profiling in a large cohort of 598 patients [115]. In an independent prospective validation cohort of 349 patients, this serum signature demonstrated strong diagnostic accuracy across all GC stages (I–IV), with an AUC of 0.86 (95% CI: 0.83–0.90). This performance surpasses conventional biomarkers such as CEA and CA19-9 and offers greater cost-effectiveness than endoscopic screening, highlighting its strong potential for clinical translation. The DESTINEX multicenter study constructed a 10-miRNA signature that distinguished GC patients from controls with AUCs of 0.958 and 0.948 in the training and validation cohorts, respectively, and notably achieved an AUC of 0.968 for detecting pT1-stage GC [116]. In a plasma-based study, *circ-KIAA1244* was significantly downregulated in GC and achieved an AUC of 0.748, with 77.42% sensitivity and 68.00% specificity for distinguishing GC from healthy controls; lower expression was also associated with lymphatic metastasis and poorer survival [117]. A systematic discovery approach identified an 8-circRNA panel that robustly distinguished GC patients from non-disease controls, with AUCs of 0.87 and 0.83 in the training and validation cohorts, respectively. Notably, this panel effectively identified early-stage GC (stage I/II) and exhibited high specificity for GC compared with other gastrointestinal cancers [5].

A multiphase study demonstrated that exosomal *lncRNA-GC1* can detect early-stage GC and monitor disease progression more effectively than conventional markers such as CEA, CA19-9, and CA72-4 [118]. A multicenter study further validated that EV-*GClnc1* shows appropriate diagnostic performance for early-stage (I/II) GC, effectively distinguishing early GC from precancerous lesions, including chronic atrophic gastritis and intestinal metaplasia [119]. In the field of early-onset GC (EOGC), a multicenter study established a 3-EV-lncRNA signature (*NALT1*, *PTENP1*, and *HOTTIP*) through genome-wide transcriptomic profiling. This signature robustly identified EOGC patients with an AUC of 0.924 in the training cohort and maintained high accuracy in two external validation cohorts (AUCs of 0.911 and 0.933). Moreover, it effectively distinguished resectable EOGC (stage I/II) and early-stage EOGC (stage I) from precancerous lesions, outperforming traditional GC-related biomarkers [120].

#### 5.3.2 Circulating RNAs as Potential Prognostic Biomarkers in GC

In addition to their diagnostic value, circulating RNAs can also serve as prognostic biomarkers. Genome-wide plasma lncRNA microarray profiling identified a panel of five circulating lncRNAs (*TINCR*, *CCAT2*, *AOC4P*, *BANCR*, and *LINC00857*) that effectively distinguished GC patients from healthy controls, with an AUC of 0.91 (95% CI: 0.88–0.95). Moreover, this lncRNA-based index decreased significantly on postoperative day 14 and correlated with tumor size, depth of invasion, lymph node metastasis, and advanced TNM stage, supporting its utility for both diagnosis and monitoring tumor dynamics [121]. Another study using a two-phase validation approach identified three plasma lncRNAs (*FAM49B-AS*, *GUSBP11*, and *CTDHUT*) that were significantly upregulated in GC patients, achieving a combined AUC of 0.818 (95% CI: 0.772–0.864). The expression levels of these lncRNAs decreased markedly on postoperative day 10, and functional assays confirmed that *FAM49B-AS* promotes GC cell proliferation and invasion, further supporting their tumor-derived origin [122]. Additionally, serum *HOXA11-AS* is significantly increased in GC patients and can distinguish GC from healthy controls with an AUC of 0.924 (95% CI: 0.881–0.967). Higher *HOXA11-AS* levels are positively associated with tumor size, TNM stage, and lymph node metastasis, while patients with lower expression exhibit better overall survival, highlighting its diagnostic and prognostic potential [123]. Integrative approaches can also identify candidate biomarkers. For example, integrative transcriptomic mining has identified *CHST14*, which may link stromal remodeling, immune infiltration, and adverse prognosis in GC [124].

Another study focusing on plasma *circERBB2*, derived from the *ERBB2* gene locus, demonstrated its potential as a prognostic biomarker. The presence of *circERBB2* in preoperative plasma was significantly associated with lymph node metastasis and poor prognosis, and high expression was identified as an independent predictor of relapse-free survival. Furthermore, longitudinal monitoring of postoperative plasma *circERBB2* levels showed potential for detecting GC recurrence [125].

#### 5.3.3 Circulating RNAs as Candidate Therapeutic Biomarkers

Circulating RNAs have been extensively investigated as therapeutic biomarkers. For example, a plasma-based five-exosomal miRNA panel (*miR-10a-5p*, *miR-25-5p*, *miR-125a-5p*, *miR-139-5p*, and *miR-450a-5p*) was developed to predict response to paclitaxel plus ramucirumab in advanced GC, with high-risk patients showing significantly shorter survival [126]. In addition, a four-circRNA panel (*hsa_circ_0009594*, *hsa_circ_0002019*, *hsa_circ_0001789*, and *hsa_circ_0003192*) was developed for the diagnosis of stage III GC. This panel achieved an AUC of 0.81 in distinguishing stage III from stage I/II disease; when combined with clinical imaging, it further improved pre-treatment diagnostic accuracy. Notably, among patients clinically diagnosed as stage III but pathologically confirmed as stage I/II, 86% were accurately identified by this circRNA-based approach, highlighting its potential to refine patient selection for neoadjuvant chemotherapy [127]. In addition, EV-*GClnc1* levels were significantly upregulated in early-stage GC and declined markedly after surgery, suggesting a tumor-specific origin. Furthermore, EV-derived *lncRNA-GC1* can predict immunotherapeutic outcomes in GC, and low levels of EV-*lncRNA-GC1* are strongly associated with an active antitumor microenvironment [128].

The above-mentioned biomarkers are listed in Table 1.

### 5.4 Critical Limitations in the Development of GC Biomarkers

Despite substantial progress in multi-omics technologies and liquid biopsy-based biomarker discovery, the clinical translation of novel biomarkers remains challenging. A key issue is that biomarker discovery and clinical utility are often not distinguished. Many studies report promising diagnostic performance with high AUC values in discovery or internal validation cohorts; however, such results do not necessarily indicate that a biomarker is ready for real-world screening, diagnosis, prognostic stratification, or treatment selection. Clinical utility requires not only statistical accuracy but also reproducibility, standardization, cost-effectiveness, prospective validation, and superiority over existing clinical approaches.

Batch effects represent a major source of technical variation, particularly in sequencing-, proteomics-, metabolomics-, and EV-based studies. Differences in sample collection time, anticoagulant type, storage conditions, freeze-thaw cycles, extraction kits, library preparation, sequencing depth, mass spectrometry settings, and bioinformatic pipelines can introduce systematic variation unrelated to true biological differences. If samples are not properly randomized and batch effects are not adequately corrected, these artifacts may artificially inflate diagnostic performance and yield biomarker signatures that fail to validate in independent cohorts. Platform heterogeneity further limits reproducibility and cross-study comparability. EV-derived biomarkers are particularly affected by differences in isolation and characterization methods, which can yield EV preparations with varying purity, yield, and levels of contamination by lipoproteins or protein aggregates. Similar concerns apply to ctDNA methylation assays, circulating nucleic acid detection, proteomic profiling, and metabolomic platforms. Even for the same biomarker, variations in assay sensitivity, normalization strategies, internal controls, and cutoff definitions may lead to inconsistent results. Reproducibility remains a major limitation in GC biomarker research. Many candidate biomarkers are identified in small, retrospective, or single-center cohorts with limited external validation, predefined diagnostic thresholds, blinded testing, or head-to-head comparisons with established indicators such as CEA, CA19-9, CA72-4, endoscopy, and histopathological diagnosis. Cohort bias may also lead to overestimation of biomarker performance. In many studies, GC patients are compared with healthy controls, whereas real-world screening populations include individuals with chronic gastritis, gastric ulcers, intestinal metaplasia, dysplasia, *H. pylori* infection, and other gastrointestinal malignancies. Tumor stage, histological subtype, Lauren classification, treatment history, ethnicity, geographic region, age, sex, and comorbidities may all influence circulating molecular profiles. As a result, a biomarker that distinguishes advanced GC from healthy individuals may not reliably detect stage I disease or differentiate early GC from premalignant lesions.

Future studies should adopt a clinically oriented translational framework. Pre-analytical procedures, assay platforms, quality control criteria, normalization methods, and bioinformatic workflows should be harmonized using unified internal controls, reference materials, and prespecified cutoff-setting strategies. Cost-effectiveness should be considered early in development, and broad multi-omics discoveries should be optimized into minimal but informative marker panels suitable for scalable clinical application. Prospective multicenter studies with predefined endpoints, locked algorithms, blinded testing, and independent validation cohorts are essential. Moreover, integration across omics layers should follow biological and clinical rationale rather than simple statistical aggregation. Genomic and epigenetic alterations may define upstream tumor evolution, transcriptomic and non-coding RNA changes may reflect regulatory programs, proteomic and metabolomic profiles may capture functional pathway activity, and EV-derived signals may represent tumor-host communication. Such biologically informed integration, combined with real-world clinical evaluation, will be essential for translating GC biomarkers from research discovery into routine clinical practice.

## 6 Emerging Therapeutic Strategies and Translational Advances in GC

The management of GC has shifted from empiric chemotherapy toward a biomarker-guided therapeutic framework. In advanced disease, routine testing for *ERBB2* (HER2), programmed death-ligand 1 (PD-L1), claudin-18.2 (CLDN18.2), and mismatch repair deficiency/microsatellite instability-high (dMMR/MSI-H) has become central to treatment selection, enabling therapy to be aligned with tumor biology rather than clinical stage alone [9,129,130]. This transition reflects the clinical implementation of molecular stratification and has redefined first-line management. A major unresolved issue, however, is biomarker co-expression, particularly in tumors that are simultaneously HER2-positive, PD-L1-positive, or CLDN18.2-positive, for which evidence-based sequencing strategies remain limited [129].

### 6.1 First-Line Biomarker-Defined Strategies

#### 6.1.1 HER2-Positive Disease

HER2-positive GC was the first molecular subtype to demonstrate a survival benefit from targeted therapy. The ToGA trial established trastuzumab plus fluoropyrimidine-platinum chemotherapy as the standard first-line regimen, improving median overall survival from 11.1 to 13.8 months [131]. In patients with HER2-positive and PD-L1-positive tumors, the addition of pembrolizumab has further improved outcomes. In KEYNOTE-811, pembrolizumab combined with trastuzumab and chemotherapy prolonged median overall survival to 20.0 months compared with 16.8 months in the control arm, supporting dual HER2 and PD-1 blockade in this subgroup [132].

#### 6.1.2 PD-L1-Positive, HER2-Negative Disease

For HER2-negative tumors with PD-L1 expression, anti-PD-1 therapy combined with fluoropyrimidine-platinum chemotherapy has become a standard first-line option. CheckMate 649 demonstrated that nivolumab plus chemotherapy improved overall survival, particularly in patients with a combined positive score (CPS) ≥ 5 [133]. Similar findings were reported in KEYNOTE-859 and RATIONALE-305, confirming that immune checkpoint inhibition has been integrated into first-line treatment across multiple clinical trial platforms [134]. Nevertheless, PD-L1 remains an imperfect biomarker due to assay variability, heterogeneous expression, and uncertainty regarding optimal cutoff thresholds [129].

However, PD-L1 should be interpreted as a context-dependent predictive biomarker rather than a simple binary indicator of benefit [135]. Its clinical utility is complicated by spatial heterogeneity, dynamic changes in expression following therapy [136,137], interobserver variability in Combined Positive Score (CPS) assessment [137], and differences among immunohistochemical assays such as 22C3, 28-8, and SP263 [138,139]. In addition, different clinical trials and regulatory settings have applied varying CPS thresholds, which may affect patient selection and cross-trial comparability [133,140,141]. Therefore, harmonized staining protocols, pathologist training, external quality assessment, and prospective validation across clinically relevant CPS cutoffs are needed to improve the reproducibility and clinical utility of PD-L1 testing in GC.

#### 6.1.3 CLDN18.2-Positive Disease

CLDN18.2 has emerged as one of the most clinically relevant novel targets in GC. As a gastric lineage-associated tight junction protein exposed during malignant transformation, it provides a biologically plausible and therapeutically actionable target [142]. The phase III SPOTLIGHT and GLOW trials demonstrated that zolbetuximab combined with chemotherapy significantly improved progression-free and overall survival in patients with CLDN18.2-positive, HER2-negative advanced disease [143,144]. These studies established CLDN18.2-directed therapy as a new first-line option and validated CLDN18.2 as a key biomarker in routine clinical practice.

#### 6.1.4 dMMR/MSI-H and EBV-Positive Disease

dMMR/MSI-H GC is characterized by a high mutational burden and marked immune infiltration, features associated with increased sensitivity to immune checkpoint blockade. Pooled analyses of pembrolizumab studies have demonstrated substantial activity in metastatic MSI-H disease [145]. In localized disease, the NEONIPIGA study reported a pathological complete response rate of 58.6% with neoadjuvant nivolumab plus ipilimumab, supporting a shift toward immunotherapy-based strategies in resectable dMMR/MSI-H tumors [146]. EBV-positive tumors also represent an immunologically distinct subgroup with frequent PD-L1 overexpression, although prospective biomarker-driven evidence remains less mature [129].

### 6.2 Second-Line and Later-Line Targeted Therapies

#### 6.2.1 Trastuzumab Deruxtecan in HER2-Positive Disease

After progression on trastuzumab-based therapy, trastuzumab deruxtecan (T-DXd) has emerged as the most important HER2-directed advance. In DESTINY-Gastric01, T-DXd significantly improved median overall survival to 12.5 months compared with 8.4 months with the physician’s choice chemotherapy [147]. Its activity is particularly relevant in GC, where HER2 expression is often heterogeneous; the antibody-drug conjugate design and bystander effect may help overcome this limitation [129]. More recent confirmatory data have further reinforced its role as the preferred subsequent HER2-directed therapy [148].

#### 6.2.2 Ramucirumab-Based Therapy in Biomarker-Unselected Disease

For patients without a targetable biomarker or after failure of biomarker-directed therapy, ramucirumab plus paclitaxel remains the standard second-line regimen. In the RAINBOW trial, this combination improved median overall survival from 7.4 to 9.6 months and also preserved functional outcomes and quality of life [149,150]. Although the absolute benefit is modest compared with some biomarker-selected therapies, ramucirumab-based treatment remains clinically important in routine practice.

### 6.3 Emerging Targets and Investigational Platforms

#### 6.3.1 FGFR2b Inhibition

FGFR2b has emerged as a promising biomarker-defined target in GC. In the phase II FIGHT trial, bemarituzumab plus mFOLFOX6 improved progression-free and overall survival in FGFR2b-selected disease [151]. Because bemarituzumab selectively targets the FGFR2b isoform, it may offer a more favorable therapeutic index than non-selective FGFR inhibition. Ongoing phase III studies will determine whether FGFR2b joins HER2 and CLDN18.2 as a standard actionable target [152].

#### 6.3.2 Novel Antigens and Antibody-Drug Conjugates

The therapeutic pipeline is expanding beyond established oncogenic drivers. TROP2-targeted antibody-drug conjugates, such as SKB264, have shown encouraging activity in previously treated GC, while *CAPRIN1*- and *DKK1*-directed strategies are also entering clinical evaluation [153,154,155]. Although these approaches remain investigational, they reflect a broader shift toward exploiting tumor-associated antigens and lineage-specific vulnerabilities.

#### 6.3.3 CLDN18.2-Directed Cellular Therapy

CLDN18.2 is also being developed as a platform for engineered immune therapies. In a phase I study, CLDN18.2-specific CAR T-cell therapy demonstrated encouraging response rates in heavily pretreated gastrointestinal cancers, supporting the feasibility of cellular immunotherapy in GC [156]. This platform extends the therapeutic relevance of CLDN18.2 beyond monoclonal antibody-based strategies.

### 6.4 Immunotherapy beyond PD-1

Resistance to PD-1 blockade has driven interest in additional immune checkpoint targets. TIGIT inhibition combined with PD-1 blockade has been extensively evaluated in GC. Although phase 2 EDGE-Gastric data and mechanistic studies supported dual TIGIT/PD-1 co-blockade [157,158], the phase 3 STAR-221 trial was discontinued in December 2025 after a futility analysis, and its final results showed no overall survival benefit with domvanalimab plus zimberelimab and chemotherapy in HER2-negative advanced gastric or gastroesophageal junction adenocarcinoma. These negative results highlight the need for biomarker-driven patient selection in TIGIT-directed combinations [159]. Likewise, combinations of checkpoint inhibitors with multikinase inhibitors such as regorafenib or lenvatinib are being explored based on their potential to modulate the tumor microenvironment and promote vascular normalization [160,161]. These studies reflect a broader strategy of overcoming immune resistance through rational combination therapy rather than simple treatment intensification.

### 6.5 Extending Precision Therapy to Operable Disease

Perioperative FLOT remains the standard backbone for fit patients with resectable locally advanced GC, following the FLOT4 trial, which demonstrated a substantial survival advantage over older epirubicin-based regimens [162]. More recently, perioperative immunotherapy has begun to reshape this setting. In MATTERHORN, the addition of durvalumab to perioperative FLOT improved 2-year event-free survival, suggesting a potential role for checkpoint inhibition beyond metastatic disease [163]. This benefit is likely to be most pronounced in biomarker-selected populations, particularly dMMR/MSI-H tumors, in which neoadjuvant immunotherapy has already demonstrated exceptionally high pathological response rates [146].

Biomarker-directed ongoing clinical trials involving targeted therapies are listed in Table 2. Despite substantial progress, several translational barriers remain. First, biomarker overlap complicates treatment prioritization in co-positive tumors [129]. Second, intratumoral and temporal heterogeneity limit the reliability of single baseline biopsies, particularly for HER2 and potentially other therapeutic markers [164]. Third, many emerging strategies still lack robust predictive biomarkers and remain in early-phase development [151,153,154,155,156]. Finally, improved integration of liquid biopsy approaches, adaptive trial designs, and biologically faithful preclinical models will be essential for refining treatment sequencing and extending precision medicine across disease stages [165,166].

### 6.6 Resistance to Biomarker-Guided Therapy

Treatment resistance significantly limits the durability and efficacy of biomarker-guided therapy. In HER2-positive GC, intrinsic and acquired resistance to trastuzumab represents a major clinical challenge. Extensive molecular profiling across tumor types has revealed that co-occurring genomic alterations in the *PIK3CA* and *KRAS* pathways can drive resistance by sustaining downstream PI3K/AKT/mTOR and RAS/MAPK signaling despite effective HER2 blockade [167]. The dual pathway co-activation phenotype underscores the importance of comprehensive genomic profiling rather than single-marker testing when evaluating HER2-targeted therapy eligibility. Furthermore, HER2 heterogeneity, both spatial within a single tumor and temporal under therapeutic pressure, also contributes to incomplete target inhibition. The emergence of HER2-low or HER2-negative clones following trastuzumab-based therapy, has been documented by longitudinal biopsy studies in the GASTHER3 trial [164]. The development of antibody-drug conjugates such as trastuzumab deruxtecan (T-DXd), which exploits the bystander killing effect to overcome heterogeneous HER2 expression, represents a rational strategy to address this limitation [147]. Nevertheless, resistance to T-DXd has also been observed, mediated by mechanisms including reduced HER2 expression, alterations in the internalization and trafficking of the ADC complex, and acquisition of mutations in the payload target. These findings collectively emphasize that effective targeting of HER2-driven GC requires not only assessing binary HER2 status but also characterizing the broader genomic context and longitudinally monitoring clonal dynamics under therapeutic pressure.

Similarly, although CLDN18.2-directed therapy with zolbetuximab has recently entered the first-line treatment landscape, the mechanisms underlying primary and secondary resistance to CLDN18.2 targeting remain poorly characterized in GC. Potential resistance mechanisms include heterogeneous CLDN18.2 expression within and between tumor lesions, clonal selection of CLDN18.2-negative or low-expressing subpopulations under therapeutic pressure, compensatory activation of alternative tight junction proteins or growth factor signaling pathways, and modulation of antibody-dependent cellular cytotoxicity (ADCC) effector function in the TME [142,143]. The long-term efficacy of CLDN18.2-targeted agents may depend on longitudinal biomarker surveillance, and the development of next-generation therapeutics such as CLDN18.2-directed ADCs, bispecific antibodies, CAR T-cell platforms and rational combination strategies.

In addition, a substantial proportion of GC patients harbor tumors that are simultaneously positive for two or more actionable biomarkers, most commonly HER2, PD-L1, and CLDN18.2, which poses a difficult clinical challenge in determining optimal treatment prioritization and sequencing [129]. Translation from molecular discovery to clinical utility therefore requires not merely identifying individual biomarkers but establishing a hierarchical framework that accounts for biomarker co-occurrence, mutual exclusivity, and context-dependent predictive value [168]. Currently, evidence-based guidelines for managing co-positive tumors are lacking, and treatment decisions are often extrapolated from single-biomarker clinical trials that systematically excluded patients with overlapping actionable alterations. However, these biomarkers frequently show complex interactions and dynamic expression. For instance, HER2 expression can be lost under therapeutic pressure, PD-L1 expression fluctuates in response to chemotherapy and inflammatory signals, and CLDN18.2 expression may vary with tumor differentiation status [136,164]. A principled clinical approach would thus integrate comprehensive baseline profiling of all actionable biomarkers, longitudinal reassessment at disease progression, and biologically informed algorithms that incorporate co-expression patterns with tumor-intrinsic biology (e.g., MSI status, tumor-mutational burden, and genomic co-alterations) to guide sequential or combinatorial therapy. The biomarker framework should function as a dynamic decisionsupport system, not a static classification tool.

## 7 Conclusion and Future Perspectives

In summary, the transition from traditional morphological classification to a deeper molecular and microenvironmental understanding has fundamentally reshaped GC research, propelled by the rapid advances in multi-omics technologies. The distinctive value of multi-omics lies in integrative interrogation rather than parallel analysis of individual molecular layers. By covering genomic, transcriptomic, proteomic, and epigenomic data via computational platforms such as IntOGen, reseachers can systermatically identify cancer driver genes, functional interactions, and tumor-specific vulnerabilities that would remain undetectable through single-layer profiling alone [169]. In GC, such multi-omics integration reveals how genetic alterations in *TP53*, *ARID1A*, and *CDH1* intersect with epigenetic and RNA modifications to shape transcriptional programs driving immune evasion and therapeutic resistance. This provides a strong biological rationale for moving beyond descriptive multi-omics cataloging toward a mechanistically informed, and clinically actionable integration paradigm. As discussed throughout this review, the advent of high-throughput multi-omics and advanced liquid biopsy technologies has generated an expanding repertoire of biomarkers for early detection, prognostic stratification, and dynamic disease monitoring. Concurrently, elucidating the complex interplay among genetic driver mutations, reversible epigenetic alterations, dynamic RNA modifications, and the tumor microenvironment has provided critical mechanistic insights into GC development and opened new opportunities for precision medicine.

Despite these advances, substantial challenges remain before these discoveries can be fully translated into routine clinical practice. The profound intertumoral and intratumoral heterogeneity of GC, together with its temporal and spatial evolution under therapeutic pressure, continues to complicate biomarker interpretation and treatment selection. In addition, methodological variability, insufficient prospective validation, and the lack of standardized clinical frameworks still limit the widespread implementation of many emerging biomarkers and targeted strategies.

Looking ahead, further progress will rely on integrating biomarker discovery with biologically grounded patient stratification and clinically actionable treatment decision-making. Large-scale prospective studies, longitudinal liquid biopsy surveillance, and the continued application of single-cell, spatial, and artificial intelligence-driven approaches are expected to refine disease classification and accelerate translational implementation. Ultimately, a deeper understanding of GC biology will be essential to enable earlier detection, more precise therapy selection, and improved survival outcomes for patients with GC.

**Table 1:** Overview of GC biomarker in this review.

Stratification		Representative Biomarkers			Current Evidence Status			Clinical Utility		
Actionable		HER2, PD-L1			Incorporated into Clinical Guidelines			Guide Diagnosis/1st-Line/2nd-Line Treatment Decisions		
Ref.	Main Finding	Circulating Biomarker(s)	Sample Type	GC Patients (N)	Control Group (N)	Control Type	Sensitivity	Specificity	AUC	95% CI
[4]	HER2 gene assessment in liquid biopsy of gastric and esophagogastric junction cancer patients qualified for surgery	HER2 gene copy number variation (CNV)	Serum (liquid biopsy)	87	40	Healthy donors	58%	98%	0.707	0.593–0.821
[170]	Prognostic value of soluble PD-L1 and exosomal PD-L1 in advanced gastric cancer patients receiving systemic chemotherapy	sPD-L1 (soluble PD-L1) + exoPD-L1 (exosomal PD-L1)	Plasma (sPD-L1) + Serum-derived exosomes (exoPD-L1)	99	N/A—prognostic study, no control group	N/A—prognostic study (all had advanced GC receiving chemotherapy)	Not applicable	Not applicable	Not applicable	N/A
**Stratification**		**Representative Biomarkers**						**Clinical Utility**		
**Near-Clinical**		**ctDNA MRD, Methylation Panels, EV-*lncRNA-GC1***						**Candidates for Recurrence Monitoring/Early Diagnosis Stratification**		
**Ref.**	**Main Finding**	**Circulating Biomarker(s)**	**Sample Type**	**GC Patients (N)**	**Control Group (N)**	**Control Type**	**Sensitivity**	**Specificity**	**AUC**	**95% CI**
[108]	Monitoring circulating tumor DNA by analyzing personalized cancer-specific rearrangements to detect recurrence in gastric cancer	ctDNA (personalized cancer-specific rearrangements)	Plasma	21	4	Nonrecurrent patients	Not applicable	Not applicable	N/A	N/A
[109]	Deep sequencing of circulating tumor DNA detects molecular residual disease and predicts recurrence in gastric cancer	ctDNA (MRD detection by deep sequencing)	Plasma	46	N/A (all patients had GC; MRD monitoring, no control group)	N/A—MRD/recurrence monitoring study	Not applicable	Not applicable	Not reported (prognostic, not diagnostic)	N/A
[107]	Circulating Tumor DNA Analysis Detects *FGFR2* Amplification and Co-occurring Resistance Mechanisms in Advanced Gastric Cancer	ctDNA (*FGFR2* amplification)	Plasma	365 (ctDNA screening); 44 paired tissue-plasma	N/A—biomarker detection study, not case-control diagnostic	N/A—detection of *FGFR2* amplification, not diagnostic	Not applicable	Not applicable	Not applicable	N/A
[118]	Circulating Exosomal Gastric Cancer-Associated Long Noncoding RNA1 as a Biomarker for Early Detection and Monitoring Progression of Gastric Cancer	*lncRNA-GC1* (exosomal)	Plasma-derived exosomes	522	85 (precancerous lesions) + 219 (healthy donors) = 304	Healthy donors; Gastric precancerous lesions	88.24%	82.29%	0.8905	0.8371–0.9438
[119]	A Liquid Biopsy Signature for the Early Detection of Gastric Cancer in Patients	EV-derived *GClnc1* (extracellular vesicle long noncoding RNA)	Plasma-derived EVs	888	158 (chronic atrophic gastritis) + 193 (intestinal metaplasia) + 501 (healthy donors) + 401 (other GI cancers) = 1253	Healthy donors; Chronic atrophic gastritis; Intestinal metaplasia; Other GI cancers	87.42% (vs. HD); 91.04% (vs. CAG); 93.02% (vs. IM); 90.71% (vs. Controls)	84.82% (vs. HD); 80.36% (vs. CAG); 74. 11% (vs. IM); 80.36% (vs. Controls)	0.9369 (vs. HD); 0.9272 (vs. CAG); 0.9101 (vs. IM); 0.9274 (vs. Controls)	0.9073–0.9664 (vs. HD); 0.8916–0.9629 (vs. CAG); 0.8714–0.9489 (vs. IM); 0.8969–0.9579 (vs. Controls)
[128]	Extracellular vesicle-derived *lncRNA-GC1* serves as a novel biomarker for predicting and monitoring the immunotherapeutic outcomes of patients with gastric cancer	EV-derived *lncRNA-GC1*	Plasma-derived EVs	760	N/A—biomarker for immunotherapy response prediction, not diagnostic	N/A—prognostic/predictive biomarker for ICI treatment outcomes	Not applicable	Not applicable	Not applicable	N/A
[110]	Detection of Gastric Cancer with Novel Methylated DNA Markers: Discovery, Tissue Validation, and Pilot Testing in Plasma	*ELMO1*, *ZNF569*, *C13orf18* (3-marker MDM panel)	Plasma	36	38	healthy controls	86%	95%	N/A	N/A
[111]	A Novel Plasma-Based Methylation Panel for Upper Gastrointestinal Cancer Early Detection	*ELMO1*, *ZNF582*, *TFPI2* (3-methylated gene panel)	Plasma	186 (UGC patients, includes gastric + other upper GI)	190	Control subjects (non-UGC)	71%	90%	0.870	0.832–0.902
**Stratification**		**Representative Biomarkers**						**Clinical Utility**		
**Validating**		**miRNA/circRNA/lncRNA Panels**						**Requires Prospective Validation**		
**Ref.**	**Main Finding**	**Circulating Biomarker(s)**	**Sample Type**	**GC Patients (N)**	**Control Group (N)**	**Control Type**	**Sensitivity**	**Specificity**	**AUC**	**95% CI**
[115]	Assessment of the Diagnostic Efficiency of a Liquid Biopsy Assay for Early Detection of Gastric Cancer	*miR-18a*, *miR-181b*, *miR-335* (3-miRNA signature)	Serum	586 (retrospective) + 349 (prospective) = 935 total	598 (discovery); normal tissue controls used in discovery	Normal tissues (discovery); no separate healthy control in validation	71.6%	87.9%	0.86	0.83–0.90
[116]	Exosomal Liquid Biopsy for the Early Detection of Gastric Cancer: The DESTINEX Multicenter Study	10-miRNA Destinex signature (8 cell-free + 9 exosomal miRNAs → final 10 overlapping)	Serum	161	102	Controls without disease	95.0% (Early stages); 86.0% (Late stages)	93.9% (Early stages); 82.9% (Late stages)	0.968 (Early stages); 0.939 (Late stages)	0.935–1.00 (Early stages); 0.907–0.972 (Late stages)
[126]	A machine-learning powered liquid biopsy predicts response to paclitaxel plus ramucirumab in advanced gastric cancer: results from the prospective IVY trial	*miR-10a-5p*, *miR-25-5p*, *miR-125a-5p*, *miR-139-5p*, *miR-450a-5p* (5-exo-miRNA panel)	Serum-derived exosomes	115 (all advanced GC receiving PTX + RAM)	N/A—predictive biomarker for treatment response (controlled vs. progressive disease)	N/A—predictive: controlled disease vs. progressive disease (not HC comparison)	66%	88%	0.84	0.76–0.92
[5]	Diagnostic efficacy of circular RNAs as noninvasive, liquid biopsy biomarkers for early detection of gastric cancer	8-circRNA panel	Serum	194	94	Non-disease controls	78.3% (training); 89.0% (validation)	78.3% (training); 62.0% (validation)	0.87 (training); 0.83 (validation)	0.82–0.93 (training); 0.77–0.90 (validation)
[114]	Diagnosis value of *miR-181*, *miR-652*, and CA72-4 for gastric cancer	*miR-181*, *miR-652*, CA72-4 (combined)	Serum	112 (60 early GC + 52 advanced GC)	50 (benign gastric lesions) + 40 (healthy controls) = 90	Gastric benign lesions; Healthy controls	92.5%	86.8%	0.917	0.856–0.975
[121]	Genome-Wide lncRNA Microarray Profiling Identifies Novel Circulating lncRNAs for Detection of Gastric Cancer	*TINCR*, *CCAT2*, *AOC4P*, *BANCR*, *LINC00857* (5-plasma-lncRNA panel)	Plasma	167	110	healthy controls	82%	87%	0.91	0.88–0.95
[122]	Genome-wide long non-coding RNAs identified a panel of novel plasma biomarkers for gastric cancer diagnosis	*FAM49B-AS*, *GUSBP11*, *CTDHUT* (3-lncRNA panel)	Plasma	173	173	Healthy controls	77.5%	73.9%	0.818	0.772–0.864
[127]	Enhancing Preoperative Diagnosis Accuracy of Stage III Gastric Cancer with Circulating circRNAs	4-circRNA panel (*hsa_circ_0001789* + *hsa_circ_0002019* + *hsa_circ_0003192* + *hsa_circ_0009594*)	Plasma	83 (16 with stage III)	67	with stage I/II	56%	89%	0.91	0.84–0.99
[120]	Diagnostic efficacy of an extracellular vesicle-derived lncRNA-based liquid biopsy signature for the early detection of early-onset gastric cancer	*NALT1*, *PTENP1*, *HOTTIP* (3-EV-lncRNA signature)	Plasma-derived EVs	43 Early-onset gastric cancer	37	Age-matched non-disease controls	82.6%	88.9%	0.924	0.889–0.953
**Stratification**		**Representative Biomarkers**						**Clinical Utility**		
**Exploratory**		**Single Proteins, Single circRNA/lncRNA**						**Requires Further Validation in Large Cohorts**		
**Ref.**	**Main Finding**	**Circulating Biomarker(s)**	**Sample Type**	**GC Patients (N)**	**Control Group (N)**	**Control Type**	**Sensitivity**	**Specificity**	**AUC**	**95% CI**
[117]	CircRNA microarray profiling identifies a novel circulating biomarker for detection of gastric cancer	*circ-KIAA1244*	Plasma	10	5	Healthy individuals	77.42%	68.0%	0.7481	0.6388–0.8573
[123]	Long noncoding RNA *HOXA11-AS* promotes gastric cancer cell proliferation and invasion	*HOXA11-AS*	Serum	94	40	Healthy controls	78.7%	97.8%	0.924	0.881–0.967
[125]	Circulating *circERBB2* as a potential prognostic biomarker for gastric cancer	*circERBB2*	Plasma	70 (preoperative); 37 had detectable *circERBB2*	N/A—prognostic study, no healthy control group	N/A—prognostic biomarker study	Not applicable	Not applicable	Not reported (prognostic, not diagnostic)	N/A
[106]	A new biomarker for the early diagnosis of gastric cancer: gastric juice- and serum-derived *SNCG*	*SNCG* (γ-synuclein)	Gastric juice + Serum	87	38 (precancerous lesions) + 44 (healthy volunteers) = 82	Gastric precancerous lesions; Healthy volunteers	95.4% (vs. control); 93.1% (vs. GPL)	86.4% (vs. control); 50.0% (vs. GPL)	0.924 (vs. control); 0.776 (vs. GPL)	0.871–0.976 (vs. control); 0.691–0.862 (vs. GPL)
[105]	The diagnostic value of serum insulin-like growth factor binding protein 7 in gastric cancer	*IGFBP7* (insulin-like growth factor binding protein 7)	Serum	169 (training) + 55 (validation) = 224	100 (training) + 55 (validation) = 155	Normal controls	36.7% (training); 34.5% (validation)	90.0% (training); 85.5% (validation)	0.774 (training); 0.758 (validation)	0.713–0.836 (training); 0.664–0.852 (validation)
[104]	*ILF2* protein is a promising serum biomarker for early detection of gastric cancer	*ILF2* (interleukin enhancer binding factor 2)	Serum	99	49 (benign gastric disease) + 51 (healthy controls) = 100	Benign gastric disease; Healthy controls	69.7% (GC vs. HC); 69.7% (GC vs. BGD); 69.7% (GC vs. BGD + HC)	91.0% (GC vs. HC); 85.7% (GC vs. BGD); 96.1% (GC vs. BGD + HC)	0.915 (GC vs. HC); 0.854 (GC vs. BGD); 0.885 (GC vs. BGD + HC)	0.873–0.957 (GC vs. HC); 0.793–0.915 (GC vs. BGD); 0.841–0.929 (GC vs. BGD + HC)

Abb:AUC, area under the curve; BGD, benign gastric disease; CAG, chronic atrophic gastritis; CI, confidence interval; CNV, copy number variation; ctDNA, circulating tumor DNA; EV, extracellular vesicle; GC, gastric cancer; GI, gastrointestinal; GPL, gastric precancerous lesions; HC, healthy control; HD, healthy donors; ICI, immune checkpoint inhibitor; IGFBP7, insulin-like growth factor binding protein 7; ILF2, interleukin enhancer binding factor 2; IM, intestinal metaplasia; lncRNA, long non-coding RNA; MDM, methylated DNA markers; MRD, molecular residual disease; N/A, not applicable; PTX, paclitaxel; RAM, ramucirumab; sPD-L1, soluble programmed death-ligand 1; UGC, upper gastrointestinal cancer.

**Table 2:** Biomarker-directed ongoing clinical trials with targeted therapy.

NCT ID	Brief Title	Phase	Enrollment (n)	Drug Combination	Contains Chemotherapy	Contains ICB	ICB Name(s)	Study Start	Primary Completion	Study Completion
NCT03563326	Intraperitoneal Infusion of EpCAM CAR-T Cell in Advanced Gastric Cancer with Peritoneal Metastasis (WCH-GC-CART)	Phase 1	40	EpCAM CAR-T cells (intraperitoneal infusion); Chemotherapy	Yes	No	NA	30 August 2018	31 December 2019	31 December 2022
NCT06821048	Study of CEA Targeting CAR-T (PTC13) in the Treatment of CEA-Positive Advanced Malignant Solid Tumors	Phase 1	18-36	FAST CEA-targeted CAR-T (PTC13); Cyclophosphamide; Fludarabine (lymphodepletion)	Yes	No	NA	24 July 2024	01 June 2026	01 December 2027
NCT07311408	SHR-1701 + Rivoceranib (± SHR-2554) in Advanced GC After First-Line Immunotherapy Failure	Phase 2	40	SHR-1701 (anti-PD-L1/TGF-β); Rivoceranib (anti-VEGFR2); ± SHR-2554 (*EZH2* inhibitor)	No	Yes	SHR-1701 (PD-L1/TGF-β dual antibody)	01 January 2026	01 January 2028	01 December 2029
NCT05942573	Continuation of Serplulimab Plus Chemotherapy After First Progression in Advanced Gastric or Gastro-oesophageal Junction Adenocarcinoma	Phase 2	107	Serplulimab (anti-PD-1); Chemotherapy (various regimens) ± Apatinib/Ramucirumab (anti-VEGFR2) ± Serplulimab (anti-PD-1)	Yes	Yes	Serplulimab	24 December 2022	01 October 2023	31 December 2024
NCT06376773	Molecular Subtype-guided Neoadjuvant Therapy for Gastric Cancer	Observational	234	Camrelizumab (anti-PD-1); Apatinib (anti-VEGFR2); neoadjuvant chemotherapy	Yes	Yes	Camrelizumab	01 June 2022	01 July 2023	30 November 2023
NCT05608785	Genotype-based First-line Treatment for Unresectable LA/Advanced Adenocarcinoma of the Stomach or Gastroesophageal Junction	Phase 1/2	45	IBI315 (anti-PD-1/HER2 bispecific antibody); TST001 (anti-Claudin18.2 monoclonal antibody); TQB2450 (anti-PD-L1 monoclonal antibody); anrotinib (anti-angiogenic tyrosine kinase inhibitor); oxaliplatin; capecitabine	Yes	Yes	IBI315 (HER2/PD-1 bispecific); TQB2450 (PD-L1 inhibitor)	01 January 2023	31 December 2023	01 October 2024
NCT06881017	Precision Targeted Therapy Based on Novel Molecular Subtyping in Advanced GC	Phase 2	140	Trastuzumab (anti-HER2); Adebrelimab (anti-PD-L1); Apatinib (anti-VEGFR2); S-1 (chemotherapy); Capecitabine (chemotherapy); Oxaliplatin (chemotherapy); Zolbetuximab (anti-Claudin18.2); SHR-A1811 (anti-HER2 ADC); SHR-A1904 (anti-Claudin18.2 ADC); SHR-1701 (anti-PD-L1/TGF-β)	Yes	Yes	Adebrelimab (PD-L1); SHR-1701 (PD-L1/TGF-β)	01 May 2025	01 August 2029	01 November 2029
NCT07257380	ctDNA-based MRD-Guided Adjuvant Therapy in LA Gastric Cancer (MRD-ATLAS)	Phase 2	90	Standard neoadjuvant therapy; Radical gastrectomy; Standard adjuvant therapy; ctDNA-MRD-guided adjuvant therapy	Yes	No	NA	30 December 2025	30 December 2029	30 December 2029
NCT05216237	Sintilimab + Apatinib + Chemo in HER-2 Negative MSS Advanced GC/GEJ	Phase 2	31	Sintilimab (anti-PD-1); Apatinib (anti-VEGFR2); Oxaliplatin + Tegafur (capeox-like chemotherapy)	Yes	Yes	Sintilimab	15 February 2022	01 June 2024	01 June 2025
NCT06415669	Paclitaxel + Apatinib + Adebrelimab in Gastric/GEJ Adenocarcinoma	Phase 2	30	Paclitaxel (chemotherapy); Apatinib (anti-VEGFR2); Adebrelimab (anti-PD-L1)	Yes	Yes	Adebrelimab	19 May 2024	19 May 2026	19 May 2027
NCT03505320	Zolbetuximab (IMAB362) plus chemotherapy and/or immunotherapy for metastatic/locally advanced unresectable or locoregional gastric/GEJ adenocarcinoma	Phase 2	143	Zolbetuximab (anti-Claudin18.2); Oxaliplatin; Leucovorin (biochemical modulator); Fluorouracil (chemotherapy); Pembrolizumab (anti-PD-1); Folinic acid (biochemical modulator); Nivolumab (anti-PD-1); Docetaxel	Yes	Yes	Pembrolizumab; Nivolumab	29 June 2018	31 July 2026	31 May 2027

Abb: ADC, antibody-drug conjugate; CAR-T, chimeric antigen receptor T-cell; ctDNA, circulating tumor DNA; GEJ, gastroesophageal junction; ICB, immune checkpoint blockade; MRD, molecular residual disease; NCT, National Clinical Trial; PTX, paclitaxel; RAM, ramucirumab; TKI, tyrosine kinase inhibitor; NA, Not Applicable.

## Data Availability

This is a review article, and no original datasets were generated or analyzed in this study. Therefore, a data availability statement is not applicable.

## Abbreviations

5-FU	5-fluorouracil
ac4C	N4-acetylcytidine
ADC	antibody-drug conjugate
ADCC	antibody-dependent cellular cytotoxicity
α-KG	α-ketoglutarate
AUC	area under the curve
BGD	benign gastric disease
BiTE	bispecific T-cell engager
CA19-9	carbohydrate antigen 19-9
CA72-4	carbohydrate antigen 72-4
CAF	cancer-associated fibroblast
CAG	chronic atrophic gastritis
CAR-T	chimeric antigen receptor T-cell
CEA	carcinoembryonic antigen
cfDNA	cell-free DNA
CI	confidence interval
CIN	chromosomal instability
circRNA	circular RNA
CLDN18.2	claudin-18.2
CNV	copy number variation
CPS	combined positive score
ctDNA	circulating tumor DNA
EBV	Epstein–Barr virus
ECM	extracellular matrix
EGC	early gastric cancer
EMT	epithelial–mesenchymal transition
EOGC	early-onset gastric cancer
EpCAM	epithelial cell adhesion molecule
EV	extracellular vesicle
FAP	fibroblast activation protein
FGFR2b	fibroblast growth factor receptor 2b
FLOT	fluorouracil, leucovorin, oxaliplatin, and docetaxel
GC	gastric cancer
GEJ	gastroesophageal junction
GGT	gamma-glutamyl transferase
GI	gastrointestinal
GPL	gastric precancerous lesion
GS	genomically stable
HC	healthy control
HD	healthy donor
HDAC	histone deacetylase
HER2	human epidermal growth factor receptor 2
HIF-1α	hypoxia-inducible factor 1α
ICB	immune checkpoint blockade
ICI	immune checkpoint inhibitor
ICOS	inducible T-cell costimulator
IFN-γ	interferon-γ
IL	interleukin
IM	intestinal metaplasia
LA	locally advanced
lncRNA	long non-coding RNA
m5C	5-methylcytosine
m6A	N6-methyladenosine
MDSC	myeloid-derived suppressor cell
miRNA	microRNA
MRD	minimal/molecular residual disease
MSI	microsatellite instability
MSI-H	microsatellite instability-high
MSS	microsatellite stable
NADPH	nicotinamide adenine dinucleotide phosphate
NET	neutrophil extracellular trap
NF-κB	nuclear factor-κB
NK	natural killer
NLR	neutrophil-to-lymphocyte ratio
PD-1	programmed cell death protein 1
PD-L1	programmed death-ligand 1
PMN	polymorphonuclear
RTK	receptor tyrosine kinase
scRNA-seq	single-cell RNA sequencing
SPEM	spasmolytic polypeptide-expressing metaplasia
ST	spatial transcriptomics
TAM	tumor-associated macrophage
TAN	tumor-associated neutrophil
TCGA	the cancer genome atlas
TGF-β	transforming growth factor-β
TIGIT	T-cell immunoreceptor with Ig and ITIM domains
TLS	tertiary lymphoid structure
TME	tumor microenvironment
TNF-α	tumor necrosis factor-α
TNM	tumor-node-metastasis
Treg	regulatory T cell
TROP2	trophoblast cell surface antigen 2
UGC	upper gastrointestinal cancer
VEGFR2	vascular endothelial growth factor receptor 2
